# Severe *Plasmodium vivax* Malaria Complicated by Secondary Hemophagocytic Lymphohistiocytosis, Disseminated Intravascular Coagulation, and Acute Pancreatitis: A Case Report

**DOI:** 10.1002/ccr3.73581

**Published:** 2026-09-28

**Authors:** Tasnim Nafian, Md. Abul Kalam Azad, Khaled Mahbub Murshed, Kazi Ali Aftab, Rubayet Shahriar, Abdullah Al Faisal

**Affiliations:** ^1^ Department of Internal Medicine Bangladesh Medical University Dhaka Bangladesh

**Keywords:** acute pancreatitis, disseminated intravascular coagulation, hemophagocytic lymphohistiocytosis, hyperferritinemia, *Plasmodium vivax*, severe malaria

## Abstract

*Plasmodium vivax* ( *P. vivax* ) malaria, once regarded as a comparatively benign infection, is increasingly recognized as a cause of severe, potentially life‐threatening multisystem disease. Concurrent secondary hemophagocytic lymphohistiocytosis (HLH), disseminated intravascular coagulation (DIC), and acute pancreatitis complicating *P. vivax* infection is exceptionally rare. A previously healthy 19‐year‐old Bangladeshi woman presented with a 5‐day history of high‐grade fever with rigors, generalized weakness, and upper abdominal pain. Laboratory evaluation demonstrated pancytopenia, markedly elevated ferritin (28,839 ng/mL), hypertriglyceridemia, hypofibrinogenemia, elevated transaminases, coagulopathy, and elevated pancreatic enzymes. Malaria rapid diagnostic testing (RDT) was positive for *P. vivax* ; peripheral blood smear was negative and PCR was unavailable. The patient fulfilled six HLH‐2004 diagnostic criteria, met International Society on Thrombosis and Hemostasis (ISTH) criteria for overt DIC, and satisfied revised Atlanta criteria for acute pancreatitis. Intravenous artesunate with multidisciplinary supportive care resulted in progressive clinical and biochemical improvement. HLH‐directed immunosuppressive therapy was not required. She was transitioned to oral artemether–lumefantrine and primaquine for radical cure and remained asymptomatic at follow‐up. This case illustrates the evolving recognition of *P. vivax* as a pathogen capable of triggering complex hyperinflammatory and multisystem complications. Early identification of underlying infection and prompt antimalarial therapy may be sufficient to achieve remission in infection‐associated HLH without immunosuppression. Clinicians in endemic regions should maintain heightened vigilance for these rare but life‐threatening complications.

## Key Clinical Message

Severe *Plasmodium vivax* malaria can rarely present with life‐threatening multisystem complications, including secondary hemophagocytic lymphohistiocytosis, disseminated intravascular coagulation, and acute pancreatitis. Early recognition of these complications and prompt antimalarial therapy targeting the underlying infection may achieve complete clinical recovery without the need for HLH‐directed immunosuppressive treatment in carefully selected patients.

## 1 Introduction

*Plasmodium vivax* is the most geographically widespread human malaria parasite, responsible for approximately 40% of malaria cases outside sub‐Saharan Africa and imposing a disproportionate burden across South Asia, including Bangladesh [1]. Historically regarded as “benign tertian malaria,” *P. vivax* has been substantially recharacterized by a growing body of evidence documenting severe and potentially fatal complications including acute liver failure, acute respiratory distress syndrome, acute kidney injury, severe anemia, and coagulopathy [2, 3]. The pathophysiological mechanisms underlying severe *P. vivax* disease encompass cytokine‐mediated immune dysregulation, endothelial activation, and microvascular dysfunction mediated by infected erythrocyte rosetting and cytoadherence [4, 5]. Hemophagocytic lymphohistiocytosis (HLH) is a life‐threatening hyperinflammatory syndrome characterized by uncontrolled macrophage and cytotoxic T‐lymphocyte activation resulting in a cytokine storm and multiorgan injury [6]. Secondary HLH most commonly arises in the context of infection, malignancy, or autoimmune disease. Malaria‐associated secondary HLH has been documented, though *P. vivax* as the precipitating trigger remains exceptionally rare [7, 8]. Disseminated intravascular coagulation (DIC) and acute pancreatitis as discrete complications of *P. vivax* have each been reported in isolated cases [9, 10]. Their simultaneous occurrence alongside secondary HLH in a single patient with *P. vivax* infection has not, to our knowledge, been previously described in the literature. We present a case of severe *P. vivax* malaria in a 19‐year‐old Bangladeshi woman complicated concurrently by secondary HLH, DIC, and acute pancreatitis, which resolved with intravenous artesunate and supportive care alone, without immunosuppressive therapy.

## 2 Case Presentation

### 2.1 Patient Information and History

A 19‐year‐old previously healthy female presented to a tertiary referral center in Dhaka, Bangladesh, with a 5‐day history of high‐grade fever with rigors and chills, generalized weakness, and upper abdominal pain associated with nausea and vomiting. She had no significant medical, surgical, or family history. She was taking no regular medications, had no history of herbal remedy use, and reported no travel outside the Dhaka metropolitan area. She was a non‐smoker and denied alcohol use. No prior history of chronic illness or autoimmune disease was elicited.

### 2.2 Physical Examination

On admission, the patient appeared acutely ill and hemodynamically unstable. Vital signs included temperature 39.8°C, heart rate 112 beats/min, blood pressure 96/60 mmHg, and respiratory rate 22 breaths/min. She was conscious, alert, and fully oriented, with a Glasgow Coma Scale score of 15/15 and no focal neurological deficit. Examination demonstrated scleral icterus, pallor, and right hypochondrial tenderness. The spleen was palpable approximately 3 cm below the left costal margin. Ascites was confirmed by positive shifting dullness. There was no lymphadenopathy or rash.

### 2.3 Investigations

Initial investigations revealed pancytopenia with severe thrombocytopenia (platelets 18 × 10^9^/L), anemia (hemoglobin 9.6 g/dL), and leukopenia (total WBC 3.3 × 10^9^/L). Markedly elevated ferritin (28,839 ng/mL), LDH (1178 U/L), and triglycerides (332 mg/dL) were noted. Liver function tests demonstrated significant transaminitis (AST 522 U/L, ALT 171 U/L), hyperbilirubinemia (total bilirubin 5.65 mg/dL; direct/conjugated bilirubin 4.49 mg/dL, indicating a predominantly direct hyperbilirubinemia consistent with hepatocellular and cholestatic injury from systemic hyperinflammation), and hypoalbuminemia (2.47 g/dL). Coagulation studies revealed a prolonged prothrombin time (19.5 s; INR 1.6), markedly reduced fibrinogen (91 mg/dL), and elevated D‐dimer (7.83 μg/mL), fulfilling ISTH criteria for overt DIC. Serum lipase (584 U/L) and amylase (262 U/L) were both elevated. Renal function was preserved (creatinine 0.49 mg/dL). The complete investigation profile is detailed in Table 1. Abdominal ultrasonography demonstrated hepatosplenomegaly, moderate ascites, bilateral mild pleural effusions, and acute nephropathy characterized by increased bilateral renal cortical echogenicity. Gallbladder wall oedema was noted without cholelithiasis or biliary ductal dilatation. Importantly, the pancreas appeared normal, helping to exclude biliary pancreatitis. Original ultrasonographic images are not available for inclusion; findings are reported as documented in the clinical record. Representative laboratory findings on admission are shown in Table 1. Absent sonographic pancreatic abnormality does not exclude acute pancreatitis, as ultrasonography has limited sensitivity in early or mild disease. Paracentesis yielded inflammatory ascitic fluid (total cell count approximately 2000 cells/mm^3^; 60% neutrophils, 40% lymphocytes), elevated protein (3.16 g/dL), and markedly elevated adenosine deaminase (ADA; 69.74 U/L). Ascitic fluid culture, malignant cytology, and GeneXpert for *Mycobacterium tuberculosis* was all negative. Although the elevated ADA raised initial concern for tuberculous peritonitis in this endemic setting, the absence of microbiological evidence, lack of chronic constitutional symptoms, mixed inflammatory rather than strongly lymphocyte‐predominant ascitic fluid, and rapid clinical resolution without antitubercular therapy indicated that the ascites represented part of the systemic inflammatory response to severe malaria complicated by secondary HLH. Malaria RDT (SD BIOLINE Malaria Ag P.f/P.v immunochromatographic assay) was positive for *P. vivax* ‐specific parasite lactate dehydrogenase antigen (Pv‐pLDH). Peripheral blood smear was negative, likely reflecting low‐density parasitemia or prior partial treatment. PCR‐based species confirmation was unavailable at our institution. Dengue serology, Widal test, Brucella serology, and blood cultures were all negative. Direct and indirect Coombs tests were negative; G6PD activity was normal (8.33 U/g Hb). The patient fulfilled six HLH‐2004 diagnostic criteria: persistent fever, splenomegaly, bicytopenia (hemoglobin and platelet lines), hyperferritinemia (28,839 ng/mL), hypertriglyceridemia (332 mg/dL), and hypofibrinogenemia (91 mg/dL). NK cell activity and soluble CD25 (sIL‐2R) assays were unavailable due to resource constraints. Retrospective application of the HScore yielded an estimated score of 264 (Table 2), corresponding to > 99% probability of reactive HLH. Coagulation findings met ISTH criteria for overt DIC. Acute pancreatitis was diagnosed per revised Atlanta criteria based on characteristic abdominal pain and serum lipase exceeding three times the upper reference limit.

**TABLE 1:** Integrated investigation profile.

Domain	Investigation	Early phase (Day 1, admission)	Intermediate (Days 2–3)	Late phase (Days 6–9)
Hematology	Hemoglobin	9.6 g/dL ↓	—	10.8 g/dL
	Total WBC	3.3 × 10^9^/L ↓	—	4.5 × 10^9^/L
	Platelet count	18 × 10^9^/L ↓	35 × 10^9^/L ↓	80 × 10^9^/L
	Peripheral smear	Pancytopenia	Pancytopenia	Bicytopenia (hemoglobin and platelet)
Inflammatory	Ferritin	28,839 ng/mL ↑	—	—
Liver function	Total bilirubin	5.65 mg/dL ↑	—	2.3 mg/dL
	Direct bilirubin	4.49 mg/dL	—	—
	AST (SGOT)	522 U/L ↑	—	—
	ALT (SGPT)	171 U/L ↑	—	—
	Albumin	2.47 g/dL ↓	—	—
Pancreatitis	Amylase	262 U/L ↑	—	—
	Lipase	584 U/L ↑	—	—
Renal	Creatinine	0.49 mg/dL	—	0.33 mg/dL
	Urea	37.5 mg/dL	—	—
Coagulation (DIC)	Prothrombin time	19.5 s ↑	—	—
	Fibrinogen	91 mg/dL ↓	—	—
	D‐dimer	7.83 μg/mL ↑	—	—
HLH criteria	LDH	1178 U/L ↑	—	—
	Triglycerides	332 mg/dL ↑	—	—
Imaging	USG abdomen	Hepatosplenomegaly, moderate ascites, bilateral mild pleural effusion; pancreas normal	—	—
Infectious	Malaria RDT/smear	Positive (*Plasmodium vivax*)	Positive	—
	Dengue IgG/IgM	—	Negative	Negative
	Blood culture	—	No growth	—
	Widal/brucella	—	Negative	—
Other	Coombs test	Negative	Negative	—
	G6PD	—	8.33 U/g Hb (normal)	—

**TABLE 2:** HScore calculation in the patient.

HScore calculation in the patient			
HScore parameter	Scoring criteria (points per category)	Max points	Patient value & score
Known underlying immunosuppression	✓ No → +0 Yes (HIV, long‐term steroids, Immunosuppressant) → +18	18	None (previously healthy) Score: +0
Temperature at presentation	< 38.4°C → +0 38.4°C–39.4°C → +33 ✓ > 39.4°C → +49	49	39.8°C Score: +49
Organomegaly	No organomegaly → +0 Hepatomegaly OR splenomegaly → +23 ✓ Hepatomegaly and splenomegaly (both) → +38	38	Hepatosplenomegaly (both present) Score: +38
Number of cytopenia (Hb, WBC, platelets)	1 lineage → +0 2 lineages → +24 ✓ 3 lineages → +34	34	Hb 9.6 ↓ Plt 18 ↓ WBC ↓ 3.3 Score: +34
Ferritin (ng/mL)	< 2000 → +0 2000–6000 → +35 ✓ > 6000 → +50	50	28,839 ng/mL Score: +50
Triglycerides (mg/dL)	< 132.7 → +0 ✓ 132.7–354 → +44 > 354 → +64	64	332 mg/dL Score: +44
Fibrinogen (mg/dL)	> 250 mg/dL → +0 ✓ ≤ 250 mg/dL → +30	30	91 mg/dL Score: +30
AST (U/L)	< 30 U/L → +0 ✓ ≥ 30 U/L → +19	19	522 U/L Score: +19
Hemophagocytosis on bone marrow biopsy	✓ No/not performed → +0 Yes → +35	35	Not performed (unavailable) Score: +0
Total *H* score			264

### 2.4 Differential Diagnosis

The differential diagnosis encompassed infectious etiologies including dengue, enteric fever, sepsis, and leptospirosis, all of which were excluded by negative serology and cultures. Hematological malignancy was not supported by the clinical picture and resolved promptly with antimalarial therapy. Autoimmune‐triggered HLH was excluded in the absence of serological or clinical evidence of systemic connective tissue disease. Biliary pancreatitis was excluded by ultrasound. Alternative causes of pancreatitis—including hypertriglyceridemia‐induced disease (fasting triglycerides were elevated in the context of HLH rather than as a primary metabolic disorder), alcohol, and drug‐induced causes—were excluded by history. A major diagnostic limitation is the absence of PCR confirmation. Although Pv‐pLDH‐based RDTs are widely used, cross‐reactivity with *Plasmodium knowlesi* has been documented; species misclassification cannot be fully excluded. To evaluate for a false‐positive RDT result, rheumatoid factor, antinuclear antibody, and hepatitis C virus antibody were tested and were all negative, arguing against rheumatoid factor interference or an autoimmune or viral mimic as the explanation for the positive Pv‐pLDH result. Additional viral workup including Epstein–Barr virus, cytomegalovirus, and HIV serology was not performed due to resource constraints. Given the positive *P. vivax* ‐targeting RDT, compatible clinical and epidemiological context, exclusion of regional alternative diagnoses, and rapid clinical improvement with antimalarial therapy alone, a presumptive diagnosis of severe *P. vivax* malaria complicated by secondary HLH, DIC, and acute pancreatitis was established. Results should therefore be interpreted as severe malaria associated with a positive *P. vivax* ‐targeting rapid diagnostic assay rather than PCR‐confirmed monoinfection.

### 2.5 Therapeutic Interventions

Intravenous artesunate (2.4 mg/kg at 0, 12, and 24 h, then once daily) was initiated promptly per WHO guidelines for severe malaria. Comprehensive supportive care included intravenous fluid resuscitation, antipyretics, and serial monitoring of hematological, hepatic, renal, and coagulation parameters. Nutritional support and bowel rest were implemented for acute pancreatitis, with gradual reintroduction of oral feeding as symptoms improved. Given clinical improvement following antimalarial initiation and attribution of HLH to *P. vivax* as the primary trigger, HLH‐directed immunosuppressive therapy (dexamethasone/etoposide) was deliberately deferred, consistent with published expert recommendations for infection‐associated HLH. Close surveillance for clinical deterioration necessitating escalation was maintained. DIC was managed conservatively; no fresh frozen plasma or platelet transfusions were required. Fever resolved by Day 5 of antimalarial therapy. Abdominal pain and nausea improved in parallel with declining pancreatic enzyme levels. Sequential laboratory investigations documented progressive normalization of thrombocytopenia, coagulopathy, ferritin, triglycerides, and transaminases (Table 3). Following clinical stabilization and restoration of oral intake, the patient was transitioned to oral artemether–lumefantrine. With confirmed normal G6PD activity, primaquine (15 mg daily for 14 days) was commenced for radical cure of hepatic hypnozoites. She was discharged on hospital Day 12 in stable condition. At outpatient follow‐up, she remained asymptomatic with fully normalized inflammatory markers and no evidence of relapse. Clinical timeline of hospital course and follow‐up is shown in Table 3.

**TABLE 3:** Clinical timeline of hospital course and follow‐up.

Time period	Clinical events and management
Pre‐admission (5 days before presentation)	Onset of high‐grade fever, rigors, generalized weakness, and upper abdominal pain. Initial evaluation and infectious workup initiated
Day 1 (admission)	Peak illness. Severe thrombocytopenia (18 × 10^9^/L), hyperferritinemia (28,839 ng/mL), hypertriglyceridemia (332 mg/dL), hypofibrinogenemia (91 mg/dL), elevated LDH (1178 U/L), hepatosplenomegaly, moderate ascites, bilateral pleural effusions. PT 19.5 s, D‐dimer 7.83 μg/mL, Malaria RDT positive (*Plasmodium vivax*). Intravenous artesunate initiated
Days 2–3	Alternative infectious workup negative (dengue, blood culture, Widal, Brucella). Coombs test negative. Three‐day course of IV artesunate completed. Step‐down to oral artemether–lumefantrine
Days 4–6	Persistent cytopenia beginning to stabilize. Supportive care continued. Fever beginning to resolve
Days 7–9	Progressive clinical and laboratory improvement. Platelet count, inflammatory markers, and coagulation profile improving. Abdominal pain and nausea resolving
Day 12 (discharge)	Clinically improved with resolving cytopenia and coagulopathy. G6PD confirmed normal; primaquine 15 mg daily for 14 days initiated for radical cure. Discharged in stable condition
Outpatient follow‐up	Asymptomatic. No clinical or laboratory evidence of relapse. Inflammatory markers fully normalized

## 3 Discussion

The traditional characterization of *P. vivax* as a “benign” infection has been substantially revised over recent decades. A systematic review by Phyo et al. [2]. Documented severe manifestations across multiple geographic settings, including severe anemia, respiratory distress, and impaired consciousness. The pathophysiological basis involves altered erythrocyte rheology from reticulocyte invasion, robust cytokine production, endothelial activation, and immune dysregulation [4]. Unlike *Plasmodium falciparum*, which sequesters in deep microvasculature, *P. vivax* ‐infected erythrocytes circulate peripherally but undergo significant rosetting and cytoadherence, contributing to microvascular dysfunction and organ injury [5]. A unique biological feature compounding the clinical burden of *P. vivax* is the formation of dormant hepatic hypnozoites, which may reactivate weeks to months after the primary episode, necessitating radical cure with an 8‐aminoquinoline agent following confirmation of adequate G6PD activity [11].

HLH represents a life‐threatening syndrome of immune dysregulation characterized by uncontrolled macrophage and cytotoxic T‐lymphocyte activation, culminating in a cytokine storm and multiorgan damage [6]. In malaria‐associated secondary HLH, persistent antigenic stimulation by parasitized erythrocytes is hypothesized to trigger aberrant lymphocyte activation and macrophage over‐activation, with dysregulated production of IL‐6, TNF‐α, IFN‐γ, and IL‐18 [12]. This cytokine milieu promotes hemophagocytosis in the bone marrow, liver, spleen, and lymph nodes, producing the characteristic cytopenia, hyperferritinemia, and hypertriglyceridemia [13] observed in this patient. Diagnosis was based on HLH‐2004 criteria, requiring fulfillment of five or more of eight defined parameters [14]. In resource‐limited settings, where NK cell activity and sIL‐2R assays are unavailable, extreme hyperferritinemia (> 10,000 ng/mL; approximately 90% sensitivity and 96% specificity for HLH [15]) serves as a pivotal diagnostic indicator. The HScore, validated as a probability calculator for reactive HLH, yielded a retrospective score of 264 (> 99% probability) in this patient, a value concordant with HScores reported in other published cases of malaria‐associated secondary HLH, in which scores have similarly clustered in the high‐probability range and correlated with the severity of hyperferritinemia, hypertriglyceridemia, and cytopenia at presentation; this consistency across geographically diverse case reports supports the HScore as a pragmatic bedside tool for identifying reactive HLH in *P. vivax* infection, particularly in resource‐limited settings where NK cell activity and sIL‐2R assays are unavailable [16]. Ferritin concentrations exceeding 20,000 ng/mL are uncommon in uncomplicated malaria and should prompt active evaluation for secondary HLH [17]. Bone marrow examination was not performed, as hemophagocytosis is neither mandatory nor pathognomonic for HLH diagnosis under HLH‐2004 criteria. The absence of lymphadenopathy, hematological malignancy, or autoimmune disease, combined with rapid resolution following antimalarial therapy, supported infection‐triggered secondary HLH.

Case reports further contextualize these findings across diverse settings. Sung et al. described a Korean man with fever, pancytopenia, and bone marrow‐confirmed hemophagocytosis who achieved complete recovery with hydroxychloroquine–primaquine alone, mirroring our patient's antimalarial‐only response [18]. Ullah et al. [19] reported HLH‐2004‐fulfilling disease resolving with intravenous antimalarials alone, without relapse. Amireh et al. [20] described cerebral malaria‐associated HLH with microangiopathic hemolytic anemia and acute kidney injury requiring artesunate‐doxycycline combination therapy. Sharma and Dawson [21] highlighted diagnostic delay in a pediatric case due to presentation rarity. Collectively, spanning South/East Asia, Africa, and pediatric‐to‐adult populations, these cases establish *P. vivax* ‐associated HLH as a reproducible, globally distributed entity where prompt antimalarial therapy determines outcome.

DIC in malaria results from a complex interplay of endothelial injury, monocyte and macrophage activation, impaired anticoagulant pathways, and fibrinolytic dysregulation, leading to widespread microvascular thrombosis with consumption of coagulation factors [9]. Although DIC is most extensively documented in severe *P. falciparum* malaria, its occurrence in *P. vivax* is increasingly recognized, attributed to systemic inflammatory response and endothelial activation. In this patient, the combination of thrombocytopenia, prolonged PT, hypofibrinogenemia, and markedly elevated D‐dimer fulfilled ISTH criteria for overt DIC [22]. The updated 2025 ISTH definition further characterizes DIC as a dynamic, acquired coagulopathy progressing from early to overt disease with hemorrhagic and thrombotic complications [17]. Management directed at the underlying infection proved sufficient; no blood product transfusions were required.

Acute pancreatitis is a rare but recognized complication of malaria, with only sporadic case reports in the literature [10]. Proposed pathophysiological mechanisms include microvascular ischemia from sequestration of parasitized erythrocytes in pancreatic capillaries and direct cytokine‐mediated pancreatic injury [5]. In this case, diagnosis was established on clinical and biochemical grounds per revised Atlanta criteria [23]: characteristic epigastric pain and serum lipase more than three times the upper reference limit. Biliary pancreatitis was excluded by ultrasonography, and alternative etiologies including alcohol, hypercalcemia, and drug‐induced pancreatitis were systematically excluded. The absence of sonographic pancreatic abnormality does not invalidate the diagnosis, as ultrasonography has limited sensitivity in mild or early pancreatitis. Recognition of malaria‐associated pancreatitis is clinically important, as abdominal pain in febrile patients in endemic regions is frequently attributed to hepatic or splenic involvement, risking diagnostic delay.

The HLH‐94 and HLH‐2004 treatment protocols, incorporating dexamethasone, etoposide, and cyclosporine, were primarily developed for primary (familial) HLH and refractory secondary HLH in hematological malignancy [14]. Current expert consensus recommends that for infection‐associated HLH, treatment of the underlying trigger is often sufficient when identified early and an effective antimicrobial agent is available [8]. In this patient, the decision to defer immunosuppressive therapy was guided by identification of *P. vivax* as the probable trigger, availability of highly effective antimalarial therapy with IV artesunate, and clinical improvement following treatment initiation. Intravenous immunoglobulin (IVIG), which is sometimes used as a lower‐toxicity bridge or adjunct in infection‐associated HLH pending resolution of the underlying trigger, was considered but not administered in this patient; given her rapid clinical and biochemical improvement on antimalarial therapy alone, the additional immunomodulatory benefit of IVIG was judged unlikely to outweigh its cost and limited availability in this setting, though it remains a reasonable option in similar cases showing a slower or incomplete response to antimicrobial therapy. This approach is consistent with published case series demonstrating resolution of malaria‐associated HLH with antimalarial therapy alone [7]. Nonetheless, close surveillance with a low threshold for escalation to HLH‐directed therapy is essential should the patient fail to respond. IV artesunate was selected as first‐line therapy per WHO guidelines for severe malaria, offering more rapid parasite clearance and improved tolerability compared with quinine [11].

Several important limitations warrant acknowledgement. Species confirmation relies solely on a positive Pv‐pLDH‐based RDT; PCR was unavailable. Cross‐reactivity with *P. knowlesi* —which can cause severe malaria—cannot be excluded [24]. Broader viral workup for alternative HLH triggers (EBV, CMV, HIV) was not performed due to resource and logistical constraints; alternative infectious contributors cannot be definitively excluded. NK cell activity, sIL‐2R, and bone marrow biopsy were unavailable. These limitations are acknowledged as important caveats to the presumptive diagnoses made herein.

## 4 Conclusions

This case documents a rare and clinically complex presentation of severe *P. vivax* malaria simultaneously complicated by secondary hemophagocytic lymphohistiocytosis, disseminated intravascular coagulation, and acute pancreatitis in a young adult female. The favorable clinical outcome achieved with prompt intravenous artesunate and multidisciplinary supportive care—without immunosuppressive therapy—reinforces the principle that effective treatment of the infectious trigger may be sufficient to induce HLH remission in selected patients with infection‐associated disease. Clinicians in *P. vivax* ‐endemic regions should maintain heightened vigilance for these rare but life‐threatening complications in patients presenting with severe malaria and disproportionate laboratory abnormalities. Extreme hyperferritinemia, coagulopathy, and elevated pancreatic enzymes should prompt systematic evaluation beyond routine malaria workup. This report should be interpreted with caution given the absence of molecular species confirmation, but it contributes to the growing literature redefining *P. vivax* as a pathogen capable of severe, multiorgan, and hyperinflammatory disease.

## Author Contributions

**Tasnim Nafian:** conceptualization, methodology, investigation, data curation, writing – original draft, writing – review and editing. **Md. Abul Kalam Azad:** supervision, project administration, validation, writing – review and editing. **Khaled Mahbub Murshed:** supervision, project administration, visualization, writing – review and editing. **Kazi Ali Aftab:** validation, formal analysis, software, writing – review and editing. **Rubayet Shahriar:** software, validation, funding acquisition, visualization, formal analysis, writing – review and editing. **Abdullah Al Faisal:** writing – review and editing, resources, project administration, visualization, formal analysis, supervision.

## Funding

The authors have nothing to report.

## Disclosure

Consent for publication was obtained from the patient.

The patient reported significant distress due to persistent fever, weakness, and the duration of her hospital admission. She expressed concern about the severity of her condition during the acute phase but felt reassured with ongoing clinical improvement. She expressed satisfaction with the care received and reported resolution of symptoms at follow‐up.

## Ethics Statement

This case report was exempted from formal ethical review per institutional guidelines for case reports. Written informed consent was obtained from the patient for publication of this case report and accompanying data.

## Conflicts of Interest

The authors declare no conflicts of interest.

## Data Availability

The data that support the findings of this study are available from the corresponding author upon reasonable request.
